# Implementation of a Cross-Layer Sensing Medium-Access Control Scheme

**DOI:** 10.3390/s17040816

**Published:** 2017-04-10

**Authors:** Yishan Su, Xiaomei Fu, Guangyao Han, Naishen Xu, Zhigang Jin

**Affiliations:** 1 School of Marine Science and Technology, Tianjin University, Tianjin 300072, China; yishan.su@tju.edu.cn; 2 School of Electrical Automation and Information Engineering, Tianjin University, Tianjin 300072, China; hanguangyao@tju.edu.cn (G.H.); xunaishen@126.com (N.X.); zgjin@tju.edu.cn (Z.J.)

**Keywords:** cross-layer, compressed sensing, medium-access control, wireless sensor networks

## Abstract

In this paper, compressed sensing (CS) theory is utilized in a medium-access control (MAC) scheme for wireless sensor networks (WSNs). We propose a new, cross-layer compressed sensing medium-access control (CL CS-MAC) scheme, combining the physical layer and data link layer, where the wireless transmission in physical layer is considered as a compress process of requested packets in a data link layer according to compressed sensing (CS) theory. We first introduced using compressive complex requests to identify the exact active sensor nodes, which makes the scheme more efficient. Moreover, because the reconstruction process is executed in a complex field of a physical layer, where no bit and frame synchronizations are needed, the asynchronous and random requests scheme can be implemented without synchronization payload. We set up a testbed based on software-defined radio (SDR) to implement the proposed CL CS-MAC scheme practically and to demonstrate the validation. For large-scale WSNs, the simulation results show that the proposed CL CS-MAC scheme provides higher throughput and robustness than the carrier sense multiple access (CSMA) and compressed sensing medium-access control (CS-MAC) schemes.

## 1. Introduction

In wireless sensor networks (WSNs), medium-access control (MAC) is an essential protocol to regulate shared channels when multiple active sensor nodes send data to the fusion center (destination). The main function of the MAC protocol is to detect, resolve, and avoid collisions because packages in collision have to be discarded and a retransmission is required.

Traditional studies on MAC mostly focused on a collision channel detection scheme where only one user is permitted to send its data at a time, such as carrier sense multiple access (CSMA) [1,2]. However, it is inefficient in checking channels status frequently, which results in serious user latency when a network consists of a great number of users.

Compressed sensing (CS) is an emerging technique for addressing sampling of sparse signals, which means that the signals are compressible in some proper basis [3,4,5]. It has now been widely used in communication filed ranging from data acquirement [6], data compression [7], and routing protocol design [8]. In WSNs, not all sensor nodes are active at the same time. It is inefficient to check channel status when only some nodes are active for transmitting data at a given time. This means the transmitted signals can be treated as a sparse vector. This phenomenon indicates that the sensor data is compressible. Therefore, CS has been used to increase the power efficiency of MAC by exploiting the correlation of the informed data to compress the transmitted data [9]. The throughput will be enhanced by adapting traditional protocol of R-ALOHA with compressed sensing to find the best user [10].

To increase the efficiency of MAC, various technologies based on CS have been proposed and can be classified into two categories: one focuses on CS-based MAC from a perspective of a physical layer [11,12,13,14], while the other relies on adding extra user identification information fields from a data link layer [15,16].

In the physical layer, by exploiting the correlation of the informed data in both time and space, CS is exploited in the MAC protocol to make the data recovery correctly [9,11]. In [12,13,14], CS methodology is used to compress the transmitter identities into data transmissions. These literatures utilize the physical layer to compress the transmitted data without the identification of active users, so that the MAC protocols become simple. However, utilizing the time and space correlation of the data in the physical layer relates the scheme to the features of the data. Moreover, a measurement matrix is frequently required, which results in high complexity and delay.

When it comes to the data link layer, there is little literature that focuses on adding extra user identification information fields in the data link layer. In [15], the sensors are equipped with a Bernoulli random generator. The data can be recovered by CS, and the Bernoulli matrix is used as a measurement matrix. However, the random binary bits of the measurement matrix results in more error in the reconstruction process of inactive node identification. In [16], compressive sensing MAC (CS-MAC) utilizes the sparsity of the request to implement a compressed request MAC scheme, to which an extra Bernoulli random matrix is added to packets. However, this literature considers this extra random matrix as the measurement matrix, which cannot represent the measurement process accurately in CS. Therefore, such a matrix decreases the accuracy in the reconstruction process.

In this paper, we attempt to combine channel state estimation in the physical layer, the actual measurement matrix, with the requests/grants in the data link layer to improve the efficiency of the MAC scheme, an attempt that has not been made before. Here, a new, cross-layer CS-MAC (CL CS-MAC) scheme, which combines the channel state of the physical layer with requests in the MAC layer to deal with the collision of the multiple transmitted packets based on compressed sensing, is proposed. The compressive complex requests are used to identify the active sensor node which makes the scheme more efficient. Moreover, because the reconstruct process is executed in the complex field of the physical layer, no bit and frame synchronizations are needed. The scheme is implemented by Universal Software Radio Peripheral (USRP) practically. It provides higher throughput and more fairness compared to the CSMA and data link layer CS-MAC schemes.

The paper is organized as follows. We introduce compressive sensing theory in Section 2 and outline the system model in Section 3. In Section 4, we discuss the design of CL CS-MAC. In Section 5, we explain the implementation of CL CS-MAC and experiments based on software-defined radio in detail. Finally, we provide concluding remarks in Section 6.

## 2. Compressed Sensing

Compressed sensing theory can reduce the rate of sampling, which makes it more efficient than the traditional sampling theory [4]. The signal $x\in R^{N\times 1}$ is an N-dimensional vector of sparsity K, which means that it has only K non-zero coefficients in this sparse signal. We can reconstruct the length N signal from M(M<N) measurements when the measurement matrix $\Phi \in R^{M\times N}$ satisfies the restricted isometry property (RIP) [17]. RIP is a related condition, which indicates the incoherence between the rows of measurement matrix and the columns of the sparse basis. As there are small numbers of activated users in a given time, the transmitted signals from all users can be considered a sparse signal in a sparse basis with an identity matrix. The measurement vector $y\in R^{M\times 1}$ is the compressed sampling vector y=Φ·x. Based on the small vector of random measurements, the sparse signal x can be recovered using a mathematical algorithm. To obtain the optimal solution, $l_{1}$-minmization is employed:

(1) $$\min{\left|\left|x\right|\right|}_{l_{1}}\text{ subject to }y=\Phi \cdot x.$$

## 3. Cross-Layer CS-MAC Design

The proposed cross-layer CS-MAC has two key ideas. First, the destination receives combined requests called “compressive complex requests”, which utilize physical layer characteristics to accurately identify active nodes and grant their transmission. Second, asynchronous requests/grants MAC can be implemented because the reconstruction process is executed in a complex field of the physical layer where there is no need for bit synchronization or frame synchronization.

### 3.1. The Cross-Layer CS-MAC Scheme

**Figure 1:**
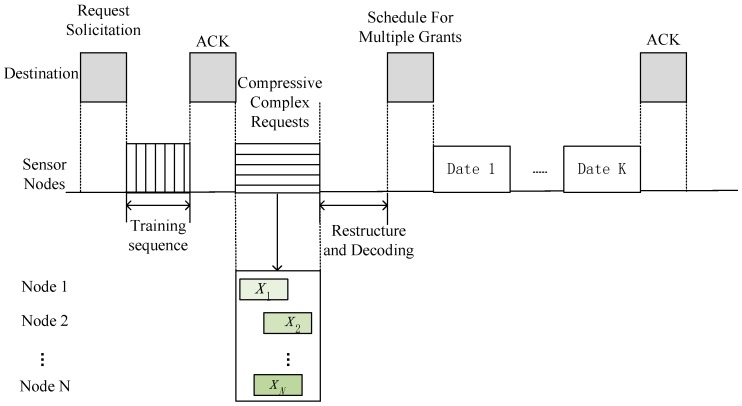
The cross-layer compressed sensing medium-access control (CL CS-MAC) scheme with asynchronous and random requests.

In a WSN system, N sensor nodes are randomly distributed and access the destination. Most of the sensor nodes remain inactive; only K nodes are active to transmit data. The destination broadcasts the request to all sensor nodes, and the sensor nodes should reply to the request so that the destination can schedule the transmission slots of these active nodes. The proposed cross-layer CS-MAC (CL CS-MAC) scheme is shown in Figure 1.

The CL CS-MAC scheme begins with the destination initiating a request solicitation. The destination enquires all the users in the system by request solicitation, querying whether the node has data to transmit. Upon receiving the solicitation, sensor nodes reply with a training sequence frame so that the destination can obtain the channel matrix, which is used as the measurement matrix in CS. After that, any active nodes randomly reply with requests, which is combined with wireless transmissions—i.e., compressive complex requests. The destination identifies active nodes and then broadcasts the schedule of active node transmission. Finally, sensor nodes transmit data according to the schedule.

### 3.2. Compressive Complex Requests during Wireless Transmission

The requests from multiple nodes are converted to modulated complex signals via constellation mapping. After matched filtering at the receiver, within each symbol duration, the observed signals at each antenna of the destination is the combination of complex signals multiplied by the channel coefficient vector. The observed signals, namely the compressive complex requests signal vector, at the $k^{th}$ antennas at the destination, can be written as

(2) $$y_{k}=\overset{N}{\underset{i=1}{\sum }}\left(h_{k,1}\cdot x_{i}\right)+n_{k}$$

where $x_{i}$ is a complex request signal of the active sensor node i. If $x_{i}$ is 0, the node does not request for the channel access. Notation $h_{k,i}$ is the channel coefficient vector from sensor node i to the $k^{th}$ antenna of the destinations, which is obtained from the training sequence. $n_{k}$ is the measurement noise.

The measurement matrix should be chosen so that it maintains minimum coherence with the basis of the sparse signal. When the channel coefficients from the sensor nodes to the *M* antennas are random and independent, the RIP is satisfied [17]. Therefore, the channel matrix can be considered as the measurement matrix. In time slot *n* during one time frame, a measurement matrix $H\in R^{M\times N}$ is determined by the channel condition. Then, the overall compressive complex requests signal at the receiver is given by

(3) y=H·x+n

where $y={\left[y_{1},y_{2},\ldots ,y_{M}\right]}^{T}$ $x={\left[x_{1},x_{2},\ldots ,x_{N}\right]}^{T}$, $n={\left[n_{1},n_{2},\ldots ,n_{M}\right]}^{T}$, and ***H*** is the channel matrix, denoted as $H=\left[\begin{array}{cccc} h_{1,1} & h_{1,2} & \ldots & h_{1,N}\\ h_{2,1} & h_{2,2} & \ldots & h_{2,N}\\ \vdots & \vdots & \ddots & \vdots \\ h_{M,1} & h_{M,2} & \ldots & h_{M,N} \end{array}\right]$.

Equation (2) indicates that the destination obtains N × M observations during one symbol duration. To obtain the measurement matrix, training sequence is used to estimate the channel condition. Sensor nodes send the training sequence $x_{0}$ to destination. The destination gets the measurement matrix based on the received sequence $y_{0}$:

(4) $$y_{0}=H\cdot x_{0}.$$

To calculate the elements in the measurement matrix, let us consider an example system consisting of four sensor nodes and a destination with two antennas. The transmission can be described as

(5) $$y_{2\times 1}=H_{2\times 4}\cdot x_{4\times 1}$$

and the measurement matrix can be defined as

(6) $$H_{2\times 4}=\left[\begin{array}{cccc} h_{11} & h_{12} & h_{13} & h_{14}\\ h_{21} & h_{22} & h_{23} & h_{24} \end{array}\right].$$

We adopt a training sequence of 00, 01, 11, and 10 to calculate the elements in $H_{2\times 4}$. The training sequence is sent from different sensor nodes in different time slots, which is also known to the destination. In a QPSK modulation scenario, the training sequence of 00, 01, 11, and 10 is modulated to $x_{1}=\left(-\frac{\sqrt{2}}{2},\;-\frac{j\sqrt{2}}{2}\right),\;x_{2}=\left(-\frac{\sqrt{2}}{2},\frac{j\sqrt{2}}{2}\right),\;x_{3}=\left(\frac{\sqrt{2}}{2},\frac{j\sqrt{2}}{2}\right),\;\;\mathrm{and}\;\;x_{4}=\left(\frac{\sqrt{2}}{2},\;-\frac{j\sqrt{2}}{2}\right)$, which form the vector $x_{4\times 1}$.

The first node sends $x_{1}$ to the destination, and the received signal at two different antennas are described as $y_{11}\;\mathrm{and}\;y_{12}$, respectively. The elements in the first column of the measurement matrix can be calculated by $h_{11}=\frac{y_{11}}{x_{1}}\;\mathrm{and}\;h_{21}=\frac{y_{12}}{x_{1}}.$ The process continues that the second node sends $x_{2}$ to the destination to calculate $h_{12}\;\mathrm{and}\;h_{22}$. Then, the third and the fourth node send $x_{3}$ and $x_{4}$ in the same way until all elements in the measurement matrix $H_{2\times 4}$ are obtained.

Under the condition that the sensor nodes are moving, the channel matrix can still be considered the measurement matrix. Because the channel coefficients from the sensor nodes to the *M* antennas of the destination are random and independent, it matches the restricted isometry property (RIP) condition. However, the obtained channel matrix might be a little different from the exact measurement matrix. Therefore, to avoid the possible error in the measurement matrix by the moving the sensors, the training sequence need to be transmitted frequently.

## 4. The Experimental Results

In order to demonstrate the practicality of the proposed CL CS-MAC scheme, we designed experiments based on software-defined radio (SDR) [18]. All experiments were conducted in an indoor environment where the surrounding objects were stationary. Nodes were in the same range as one another and transmitted with the same power level. In the experiments, a universal software radio peripheral (USRP) connected to a laptop running GNU Radio acted as a node. The transmission frequency was 2.45 GHz with GMSK modulation. The transmission power of the node was changed from −48 to −28 dBm. The noise power was −60 dBm. We randomly chose K nodes to send requests for accessing the channel.

**Figure 2:**
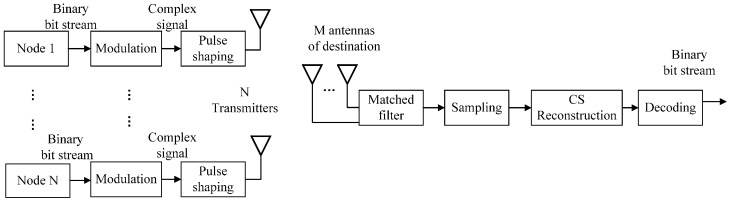
The structure of sensor nodes and destination.

The signal transmission process is illustrated in Figure 2. Some modulation technology can be selected in the GNU radio. In this paper, GMSK is selected in the experiment. Active sensor nodes send their data to the destination. To obtain enough measurement values, the destination is equipped with *M* antennas so that every signal of the sensor node has *M* degrees of freedom.

### 4.1. Determination of Active Nodes

**Figure 3:**
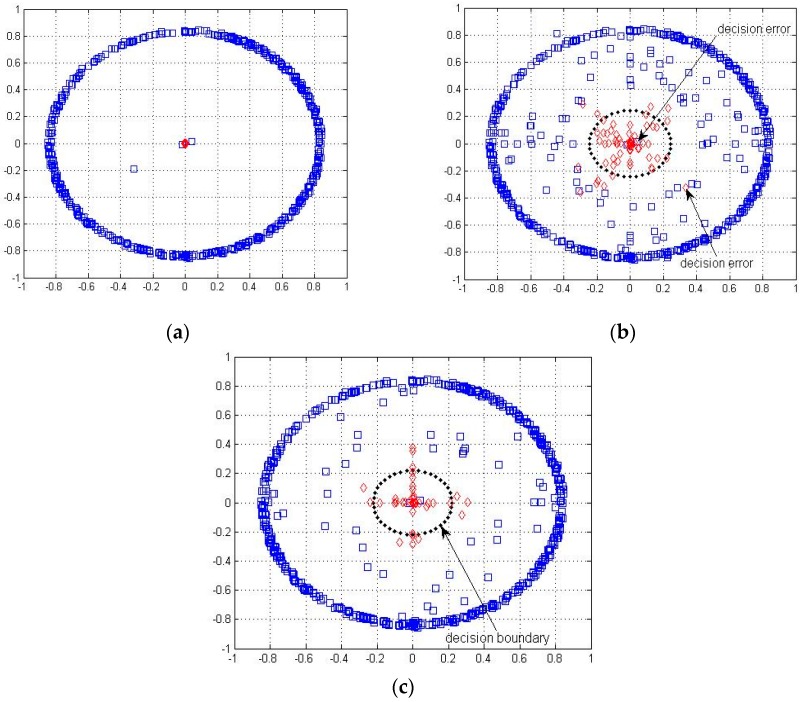
The symbol constellation diagram. (**a**) Original symbol constellation. (**b**) Recovery symbol constellation (signal/noise ratio (SNR) = 16 dB). (**c**) Recovery symbol constellation (SNR = 28 dB).

We used recovery symbol constellation diagrams at the destination to determine whether the node was active or not. The constellation diagrams of 500 symbols at the sensor nodes and at the destination are shown in Figure 3. Figure 3a is the original symbol constellation, where red diamond markers denote the inactive nodes and blue square markers denote the active nodes.

Figure 3b,c show the recovery constellations of reconstructed symbols at the destination with different signal/noise ratios at the transmitters. It can be seen that reconstructed symbols are more concentrated near the center with a higher SNR. The inner black circle is the decision boundary, and the red dots represent the symbol of inactive nodes. The red dot symbol inside the decision boundary is considered as the inactive node, while the one outside the decision boundary is considered as the active node where the decision error occurs. In order to decrease decision error such that the inactive node is considered as active one, the training sequence is used to determine the boundary. Moreover, the training sequence is used to estimate the channel matrix. The furthest distance between zero and all reconstructed training sequence symbols of inactive nodes is set as the decision boundary, as indicated by the dotted line in Figure 3b,c.

If most recovery symbols are located inside the decision boundary, the sensor node is considered inactive. According to this rule, we found that, with lower SNR, although there are more red diamond markers appearing outside the boundary, we can still correctly decide which node is inactive. It proves the robustness of this scheme.

### 4.2. Identification of Active Nodes

To identify active nodes correctly, in this experiment, active nodes sent 2000 binary bit streams to the destination. At the destination, after acquiring the measurement matrix by the training sequence, we sampled the modulated complex symbol to reconstruct the original signal. By comparing the original binary bit and the reconstructed binary bit, we can get the exact recovery binary bit rate.

**Figure 4:**
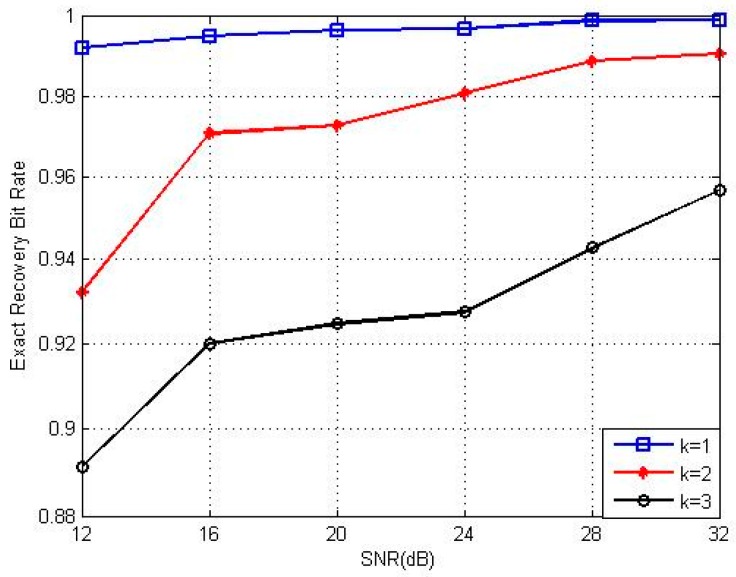
Recovery performance under different SNRs at different K values.

**Figure 5:**
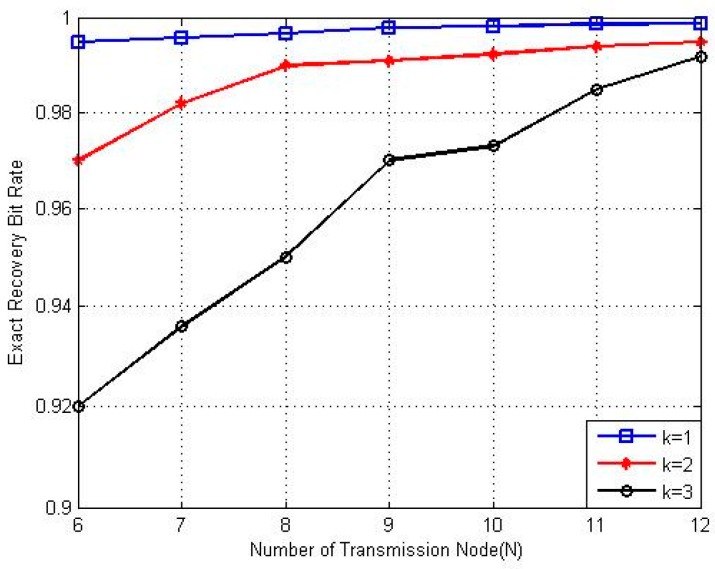
Recovery performance with transmission node (N).

**Figure 6:**
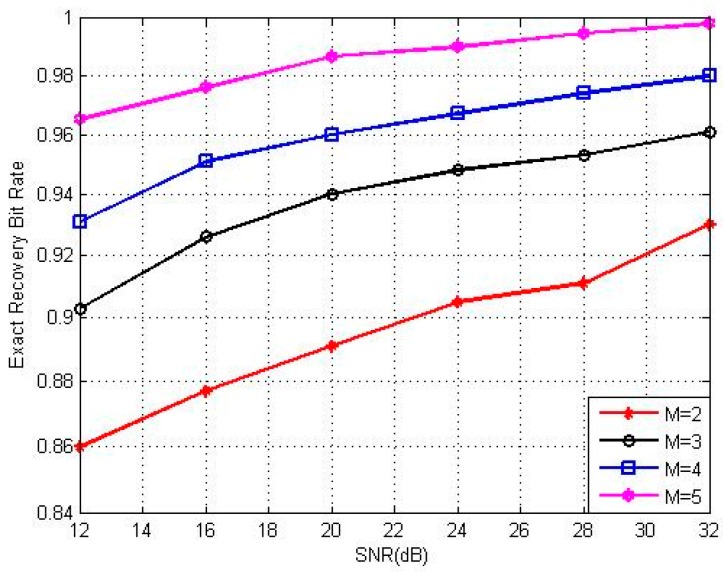
Recovery performance with receiving antennas *M*.

Figure 4, Figure 5 and Figure 6 show the exact recovery bit rate of the experiments with different parameters. The exact recovery bit rate with sparse degrees versus the transmitted SNR of the sensor nodes is shown in Figure 4. The exact recovery bit rate of transmission will increase as the SNR increases.

Figure 5 shows the results of the exact recovery bit rate with different total transmission node (N) values. As N increases, the system becomes sparser. It can be seen that the sparser the system is, the higher the exact recovery bit rate will appear to be. The performance improves when there are more inactive nodes.

We show the relationship between the number of receiving antennas *M* and the exact recovery bit rate in Figure 6. The four lines in Figure 6 indicate the exact recovery bit rate with different receiving antennas *M*. We found that the largest *M* system has the highest exact recovery bit rate, which means that a large number of receiving antennas will be beneficial for the correct transmission of the system. This is because the more receiving antennas there are, the more measured values will be received.

## 5. Simulation Performance of Cross-Layer CS-MAC Scheme

In order to test the performance of the proposed scheme with a large number of nodes, we simulated and compared different performances under CL CS-MAC, CS-MAC, and CSMA schemes. In the CSMA scheme, the packet in a collision should be lost, while in CL CS-MAC and CS-MAC, the compressive sensing is utilized to avoid the collision, and the concurrent signal can be departed by compressive sensing. However, CS-MAC only deals with the random binary matrix as the measurement matrix and cannot accurately match the measurement process, especially with low sparsity and low SNR. This results in high decision error in exact active node identification. Therefore, collisions cannot be efficiently avoided.

**Figure 7:**
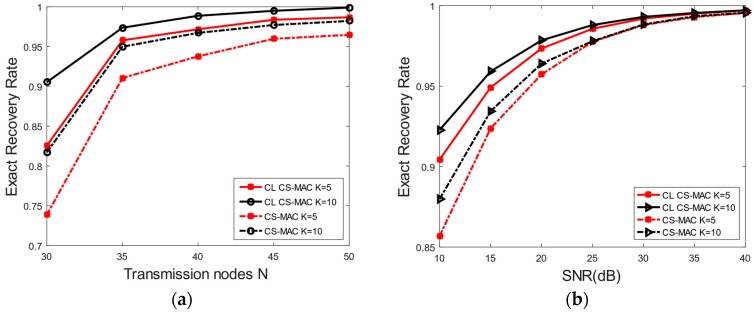
Software simulation of exact recovery rate. (**a**) Exact recovery rate of transmission nodes; (**b**) Exact recovery rate of different SNR.

**Figure 8:**
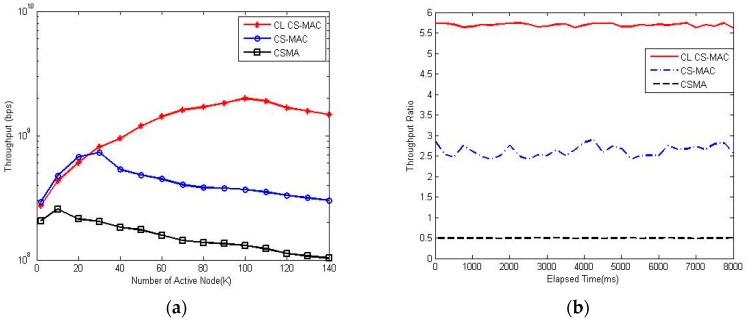
Software simulation result of throughput. (**a**) The throughput of different schemes; (**b**) The throughput ratio of different schemes.

Figure 7 shows the comparison of the exact recovery rate of the CL CS-MAC and CS-MAC with the transmission node number and the SNR. It can be seen that the exact recovery rate of CL CS-MAC is higher than that of CS-MAC with the same transmission nodes and SNR. The same trend is also shown in Figure 8, where the exact recovery rate of CL CS-MAC is higher than that of CS-MAC with the same SNR. This means that the CL CS-MAC scheme can identify the exact active node more accurately than the CS-MAC scheme.

Figure 8 shows the throughput of different schemes versus the number of active nodes. For the CSMA scheme, as the number of active node increases, the channel becomes more congested and collision occurs more frequently, so the throughput of the CSMA scheme declines. For CS-MAC and CL CS-MAC, the throughput can rise steeply as the number of active node increase. However, as the active node continues to grow, the throughput will slightly decline. The critical point of the number of active nodes of CL CS-MAC is much larger than that of CS-MAC. Further, the throughput ratio of CL CS-MAC is better than that of CS-MAC. The reason is that the CL CS-MAC scheme can identify the exact active node more accurately than can the CS-MAC scheme.

## 6. Conclusions

We proposed a Cross-Layer compressive sensing medium-access control (CL CS-MAC) scheme by considering the wireless transmission of physical layer as a compress process of request packets in MAC layer. The asynchronous and random requests scheme is implemented without synchronization payload by software-defined radio (SDR) practically. We used compressive complex requests to identify active sensor nodes. The experiment and simulation results indicate that the proposed CL CS-MAC scheme provided higher throughput and robustness than CSMA and CS-MAC schemes.
